# Longitudinal ECG changes among adults with HIV in Tanzania: A prospective cohort study

**DOI:** 10.1371/journal.pgph.0002525

**Published:** 2023-10-25

**Authors:** Faraan O. Rahim, Francis M. Sakita, Lauren Coaxum, Amedeus V. Maro, James S. Ford, Kate Hatter, Kalipa Gedion, Saad M. Ezad, Sophie W. Galson, Gerald S. Bloomfield, Alexander T. Limkakeng, Monica S. Kessy, Blandina Mmbaga, Julian T. Hertz

**Affiliations:** 1 Duke Global Health Institute, Duke University, Durham, North Carolina, United States of America; 2 Kilimanjaro Christian Medical Centre, Moshi, Tanzania; 3 Kilimanjaro Christian Medical University College, Moshi, Tanzania; 4 Department of Emergency Medicine, Duke University School of Medicine, Durham, North Carolina, United States of America; 5 Department of Emergency Medicine, University of California, San Francisco, San Francisco, California, United States of America; 6 British Heart Foundation Centre of Research Excellence and NIHR Biomedical Research Centre at the School of Cardiovascular and Metabolic Medicine and Sciences, King’s College London, London, United Kingdom; 7 Department of Internal Medicine, Duke University School of Medicine, Durham, North Carolina, United States of America; 8 Benjamin Mkapa Hospital, Dodoma, Tanzania; University of Embu, KENYA

## Abstract

The prevalence of cardiovascular disease (CVD) is rising among people with HIV (PWH) in sub-Saharan Africa (SSA). Despite the utility of the electrocardiogram (ECG) in screening for CVD, there is limited data regarding longitudinal ECG changes among PWH in SSA. In this study, we aimed to describe ECG changes over a 6-month period in a cohort of PWH in northern Tanzania. Between September 2020 and March 2021, adult PWH were recruited from Majengo HIV Care and Treatment Clinic (MCTC) in Moshi, Tanzania. Trained research assistants surveyed participants and obtained a baseline ECG. Participants then returned to MCTC for a 6-month follow-up, where another ECG was obtained. Two independent physician adjudicators interpreted baseline and follow-up ECGs for rhythm, left ventricular hypertrophy (LVH), bundle branch blocks, ST-segment changes, and T-wave inversion, using standardized criteria. New ECG abnormalities were defined as those that were absent in a patient’s baseline ECG but present in their 6-month follow-up ECG. Of 500 enrolled participants, 476 (95.2%) completed follow-up. The mean (± SD) age of participants was 45.7 (± 11.0) years, 351 (73.7%) were female, and 495 (99.8%) were taking antiretroviral therapy. At baseline, 248 (52.1%) participants had one or more ECG abnormalities, the most common of which were LVH (n = 108, 22.7%) and T-wave inversion (n = 89, 18.7%). At six months, 112 (23.5%) participants developed new ECG abnormalities, including 40 (8.0%) cases of new T-wave inversion, 22 (4.6%) cases of new LVH, 12 (2.5%) cases of new ST elevation, and 11 (2.3%) cases of new prolonged QTc. Therefore, new ECG changes were common over a relatively short 6-month period, which suggests that subclinical CVD may develop rapidly in PWH in Tanzania. These data highlight the need for additional studies on CVD in PWH in SSA and the importance of routine CVD screening in this high-risk population.

## Introduction

Since the start of the HIV epidemic in the early 1980s, approximately 84.2 million people have been infected with HIV and 40.1 million have died from its complications worldwide [1]. Although the advent of effective antiretroviral therapy (ART) in the mid-1990s has greatly reduced HIV-related deaths and extended the life expectancy of people with HIV (PWH), the burden of non-communicable diseases (NCD) in this population has increased in the past two decades [2]. In particular, cardiovascular disease (CVD) has become one of the leading causes of morbidity and mortality among PWH, accounting for roughly 2.6 million disability-adjusted life-years globally per year [3]. Multiple studies have demonstrated that PWH are at an increased risk of myocardial infarction [4, 5], cardiomyopathy [6], heart failure [7, 8], pulmonary hypertension [9, 10], ischemic stroke [11, 12], arrhythmias [13, 14], and sudden cardiac death [15]. Chronic inflammation and cardiometabolic side effects of antiretroviral therapy (ART) have been implicated as possible mechanisms underlying the association between HIV and CVD [16].

The 12-lead electrocardiogram (ECG) is a non-invasive clinical tool that can be used to screen for CVD among PWH. In resource-rich settings, certain ECG abnormalities, including ST/T abnormalities [17, 18], atrial fibrillation/flutter [19, 20], and QT interval prolongation [18, 21–24], have been described as more common among adults with HIV than those without HIV. Several studies in these settings have also observed a number of longitudinal ECG changes among HIV-infected patients taking ART [25–28]. In sub-Saharan Africa (SSA), which suffers the largest burden of HIV globally [29], less is known about the prevalence of ECG abnormalities among PWH. Although a few studies from Nigeria, Cameroon, Uganda, and Tanzania have reported a higher prevalence of prolonged QTc intervals, ST segment deviations, ventricular hypertrophy, T-wave inversion, and Q waves in PWH relative to HIV-negative controls [30–37], they have all employed cross-sectional designs. To our knowledge, no studies have assessed longitudinal ECG changes among PWH in SSA.

As improved HIV care has resulted in extended life expectancies, PWH in SSA are increasingly likely to succumb to CVD, which may be detected by routine ECG screening. However, the incidence of pathologic ECG changes among PWH in SSA is currently unknown. In this study, we aimed to prospectively describe longitudinal ECG changes over a 6-month period among PWH in Tanzania.

## Methods

### Setting & recruitment

This study was conducted at the Majengo HIV Care and Treatment Clinic (MCTC), a government-funded HIV clinic that serves approximately 1200 adults across the urban center and surrounding rural districts of Moshi, Tanzania. The facility offers free outpatient HIV-related care that includes providing ART and routine follow-ups. All adult patients (age ≥ 18 years) who presented to MCTC for routine HIV care during the enrollment phase between September 1, 2020 and March 1, 2021 were eligible for this study. No other exclusion criteria restricted participation. Trained research assistants offered enrollment to patients at MCTC during clinic check-in and obtained written informed consent from interested participants.

### Study procedures

Research assistants administered a standardized survey to all participants. This survey, which was based on the World Health Organization (WHO) STEPS tool for NCD risk factor surveillance [38], gathered information about sociodemographic background, medical history, and lifestyle behaviors from each enrolled participant. During the initial enrollment, research assistants also measured participant height, weight, and blood pressure. Point-of-care glucose was measured using a glucometer (GlucoPlus Blood Glucose Monitoring System, GlucoPlus, Montreal, Canada), and participants were questioned whether they had consumed anything earlier in the day to differentiate between fasting and random blood glucose levels. Additionally, research assistants acquired a resting twelve-lead ECG from all participants using the tablet-based PADECG (Edan Instruments, Shenzhen, China). These research assistants received hands-on training from a physician (JTH) on ECG acquisition and had several years of experience obtaining ECGs for research studies.

Information about HIV care, such as time since diagnosis, CD4 count, HIV viral load, and past and current ART use, was gathered from participants’ MCTC medical records. If CD4 count and/or HIV viral load were not documented in the medical record, a blood sample was obtained from the participant and tested at the Kilimanjaro Clinical Research Institute Biotechnology Laboratory. CD4 count assays were performed using the BD FACSCount and BD FACSCalibur instruments (BD Biosciences, Franklin Lakes, New Jersey), and HIV viral load assays using the Abbott m2000 Reverse Transcriptase instrument (Abbott Laboratories, Chicago, Illinois).

### Six-month follow-up

Participants were asked to return to MCTC six months after the initial enrollment. These follow-up visits were conducted from March 1^st^, 2021 through September 2^nd^, 2021. They scheduled to coincide with participants’ regular clinical follow-up appointments at MCTC, which typically occur at 3- or 6-month intervals. During the visit, research assistants re-assessed participant blood pressure and acquired a follow-up 12-lead ECG. Participants who died or were otherwise lost to follow-up were excluded from statistical analyses.

### Study definitions

In this study, history of hypertension, diabetes, myocardial infarction (MI), stroke, heart failure, and chronic kidney disease (CKD), were determined based on participant self-report. Obesity was defined as measured body mass index (BMI) ≥30 kg/m^2^, as per CDC guidelines [39], and sedentary lifestyle as participant self-report <150 minutes of moderately vigorous exercise per week, as per WHO guidelines [40]. Elevated blood pressure was defined as measured systolic blood pressure ≥140 mmHg or measured diastolic blood pressure ≥90 mmHg at enrollment. Point-of-care glucose was considered a fasting value if the participant reported consuming nothing earlier in the day apart from water; otherwise, it was considered random. Hyperglycemia was defined per the American Diabetes Association as fasting glucose >7.0 mmol/L or random glucose >11.1 mmol/L [41]. Virologic suppression was defined as most recent HIV RNA viral load <200 copies/mL. Lifestyle behaviors, including alcohol and tobacco use, were determined based on participant self-report.

### ECG parameter definitions

Several ECG parameters were also defined for this study. The PADECG machine automatically measured heart rate (HR), PR, and QRS intervals for each ECG. It also calculated QTc interval using the Bazett formula. Resting tachycardia was defined as machine-measured HR ≥100 beats per minute. Prolonged and short PR interval were defined as >200 ms and <120 ms, respectively. Prolonged QTc interval was defined as ≥450 ms for males and ≥460 ms for females, in accordance with international guidelines [42]. Left ventricular hypertrophy (LVH) was defined based on Sokolow-Lyon Criteria [43]. Bundle branch block was defined as QRS width >120 ms with a supraventricular rhythm; complete left bundle branch block (LBBB) and complete right bundle branch block (RBBB) were defined by American Heart Association guidelines [44]. ST elevations, ST depressions, and T-wave inversion was defined by Fourth Universal Definition of Myocardial Infarction guidelines [45]. Specifically, ST elevation was defined as ≥1 mm of ST elevation at the J-point in two contiguous leads except in leads V2-V3, where the cutoffs are ≥2 mm in men ≥40 years, ≥2.5 mm in men <40 years, and ≥1.5 mm in women. ST depression was defined as horizontal or downsloping ST depression ≥0.5 mm in two contiguous leads. T-wave inversion was defined by T wave inversion ≥1 mm in two contiguous leads. Atrial and ventricular ectopy were defined as at least one premature atrial contraction and at least one premature ventricular contraction, respectively.

### ECG interpretation

Initial and six-month ECGs were interpreted by at least two independent physician adjudicators (JTH, LC, JSF, KH, KGK, SME, FMS, AVM, SWG), who were trained in either emergency medicine or cardiology. Adjudicators were blinded to all clinical data other than participant age and sex and were tasked with identifying rhythm, LVH, bundle branch blocks, ST elevation, ST depression, T-wave inversion, atrial ectopy, and ventricular ectopy in each ECG using the criteria outlined above. To ensure consistency in interpretation between baseline and follow-up ECGs, both the baseline and follow-up ECG for a given participant were interpreted by the same pair of adjudicators. Agreement among physician adjudicators on LVH (95.1%), bundle branch blocks (99.4%), ST elevation (94.3%), ST depression (95.2%), atrial ectopy (93.8%), and ventricular ectopy (99.8%) was excellent. In cases of disagreement, a third physician adjudicator served as the tiebreaker.

### Statistical analyses

The primary purpose of this study was to identify longitudinal changes between baseline and 6-month follow-up ECGs in a cohort of PWH in Tanzania. All statistical analyses were performed in the R suite. Categorical variables are presented as proportions, and continuous variables as averages with standard deviation. Sample size calculations for this study have been previously reported [36]. Participants who received a baseline but not a follow-up ECG were excluded from analysis. Additional missing data were recorded and similarly excluded. BMI was calculated directly from measured height and weight. Tanzanian shillings (TSH) were converted to United States dollars (USD) using the World Bank 2021 conversion rate of 1 USD = 2297.76 TSH [46]. New resting tachycardia, T-wave inversion, LVH, prolonged QTc, bundle branch block, short PR, prolonged PR, ST elevation, ST depression, atrial ectopy, and ventricular ectopy were defined by the presence of any of these ECG abnormalities on a follow-up ECG that were not present on baseline ECG for the same participant. Univariate associations between baseline participant characteristics and the presence of any new ECG abnormality at 6-month follow-up were assessed via Pearson’s chi-squared (for categorical variables) and Student’s t-test (for continuous variables). Univariate odds ratios were calculated directly from two-by-two contingency tables. Multivariate logistic regression was then performed to determine the independent association of each predictor on the development of new ECG abnormalities at 6-month follow-up. Any variable with possible univariate association with new ECG abnormalities (p<0.5) was included in the multivariate model, along with participant age and sex.

### Ethics statement

The Tanzania National Institute for Medical Research, the ethics committee at Kilimanjaro Christian Medical Centre in Tanzania, and the institutional review board at Duke Health all reviewed this research study and granted ethical approval. All participants provided written, informed consent prior to enrollment. All actionable clinical findings were shared with the primary clinical team at MCTC. The data collected from this study are available from the corresponding author on reasonable request.

## Results

**Fig 1:**
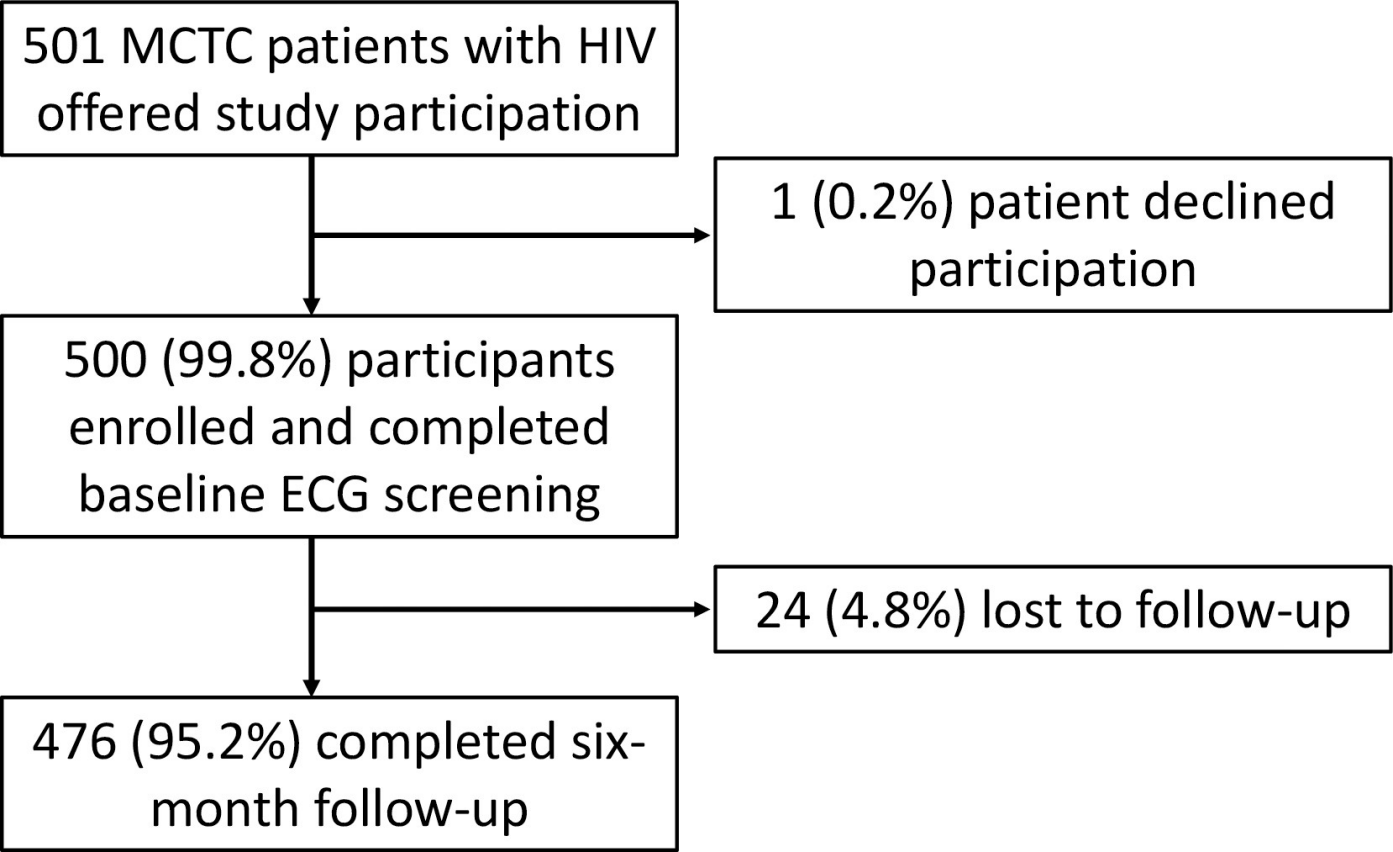
MCTC participant recruitment and follow-up flow diagram.

During the enrollment phase, 501 eligible MCTC patients were offered study participation, and 500 (99.8%) consented and were enrolled. All 500 participants completed the survey, underwent baseline ECG screening, and were asked to return for follow-up assessment in 6 months. A total of 476 (95.2%) participants completed follow-up and were therefore included in the present analysis; 24 (4.8%) participants were lost to follow-up and therefore excluded (**Fig 1**).

**Table 1** presents the baseline demographics, HIV-related parameters, self-reported medical comorbidities, and lifestyle behaviors of the 476 study participants. The majority of participants (n = 351, 73.7%) were female, and the mean age was 45.7 (± 11.0) years. Nearly all (n = 495, 99.8%) were taking some form of ART; the mean duration of ART therapy was 5.1 (± 3.7) years, and 455 (95.6%) participants had achieved HIV virologic suppression. Ninety-six participants (20.2%) were obese, 151 (31.7%) presented with a measured elevated blood pressure, and 16 (3.4%) had measured hyperglycemia. Fifty-five (11.6%) self-reported a history of hypertension, 9 (1.9%) self-reported a history of diabetes, and none reported a known history of MI. About half of participants (234, 49.2%) reported current alcohol use, and 45 (9.5%) reported current tobacco use.

**Table 1:** Characteristics of participants presenting for routine HIV care at MCTC (N = 476).

Characteristic	n	%
**Demographics**		
Sex		
Male	125	26.3
Female	351	73.7
Age, mean (sd), years	45.7 (11.0)	
Highest level of education attained		
None	30	6.3
Primary	334	70.2
Secondary	88	18.5
University	24	5.0
Income, mean (sd), USD^a^	43.45 (65.00)	
Tribal affiliation		
Chagga	258	54.2
Pare	61	12.8
Sambaa	23	4.8
Masaai	9	1.9
Meru	7	1.5
Other	118	24.8
**HIV-Related Parameters**		
HIV Virologic Suppression (<200 copies/mL)	455	95.6
CD4, mean (sd), cells/mm^3^	487.7 (263.1)	
Years since HIV diagnosis, mean (sd)	5.7 (4.2)	
Current ART		
TDF/lamivudine/dolutegravir	467	98.1
Other	8	1.7
None	1	0.2
History of protease inhibitor exposure	13	2.7
History of abacavir exposure	14	2.9
Duration of ART therapy, mean (sd), years	5.1 (3.7)	
**General Health Parameters**		
Obesity	96	20.2
BMI, mean (sd), kg/m^2^	25.3 (5.1)	
Elevated blood pressure	151	31.7
Hyperglycemia	16	3.4
**Self-reported Medical Comorbidities**		
Self-reported history of hypertension	55	11.6
Self-reported history of diabetes	9	1.9
Self-reported history of MI	0	0
Self-reported history of stroke	5	1.1
Self-reported history of heart failure	1	0.2
Self-reported history of chronic kidney disease	2	0.4
Self-reported family history of MI or stroke	93	19.5
**Lifestyle/Health Behaviors**		
Current alcohol use	234	49.2
Current tobacco use	45	9.5
Sedentary Lifestyle	154	32.4
Daily fruit and vegetable consumption	59	12.4

^a^Data unavailable for 99 participants

The baseline ECG characteristics of study participants are presented in **Table 2**. All participants were in sinus rhythm at initial enrollment. A total of 248 (52.1%) participants were noted to have ECG abnormalities at baseline, including 108 (22.7%) with LVH, 89 (18.7%) with T-wave inversion, 58 (12.2%) with atrial ectopy, 47 (9.9%) with ST elevation, 20 (4.2%) with prolonged PR interval, 17 (3.6%) with prolonged QTc interval, 15 (3.2%) with resting tachycardia, 7 (1.5%) with short PR interval, 4 (0.8%) with bundle branch block, and 3 (0.6%) with ST depression. No patients presented with baseline ventricular ectopy.

**Table 2:** Baseline ECG abnormalities of study participants.

Baseline ECG Abnormality	n	%
Left Ventricular Hypertrophy	108	22.7
T-Wave Inversion	89	18.7
Atrial Ectopy	58	12.2
ST Elevation	47	9.9
Prolonged PR	20	4.2
Prolonged QTc	17	3.6
Resting Tachycardia	15	3.2
Short PR	7	1.5
Bundle Branch Block	4	0.8
LBBB	2	0.4
RBBB	2	0.4
ST Depression	3	0.6
Non-Sinus Rhythm	0	0
Ventricular Ectopy	0	0
Total with baseline ECG abnormality	248	52.1

Overall, 112 (23.5%) of study participants developed a new ECG abnormality at six-month follow-up (**Table 3**). The most common new ECG abnormality was new T-wave inversion (n = 40, 8.4%). Other new ECG abnormalities observed included LVH (n = 22, 4.6%), ST elevation (n = 12, 2.5%), prolonged QTc (n = 11, 2.3%), prolonged PR (n = 11, 2.3%), resting tachycardia (n = 10, 2.1%), ST depression (n = 10, 2.1%), short PR (n = 5, 1.1%), atrial ectopy (n = 5, 1.1%), and ventricular ectopy (n = 2, 0.4%). No participants developed new arrhythmia or bundle branch block.

**Table 3:** New ECG abnormalities in study participants at 6-month follow-up.

New ECG Abnormality	n	%
T-Wave Inversion	40	8.4
Left Ventricular Hypertrophy	22	4.6
ST Elevation	12	2.5
Prolonged QTc	11	2.3
Prolonged PR	11	2.3
ST Depression	10	2.1
Resting Tachycardia	10	2.1
Short PR	5	1.1
Atrial Ectopy	5	1.1
Ventricular Ectopy	2	0.4
Non-Sinus Rhythm	0	0
Bundle Branch Block	0	0
LBBB	0	0
RBBB	0	0
Any new ECG abnormality	112	23.5

On univariate analysis, there were no associations between patient characteristics and the development of new ECG abnormalities (**Table 4)**. Multivariate logistic regression also did not reveal any baseline predictors of 6-month ECG changes (**S1 Table**).

**Table 4:** Associations between participant characteristics and new ECG abnormalities at 6-month follow-up (N = 476).

Participant Characteristic	One or more New ECG Abnormalities (N = 112) n (%)	No New ECG Abnormalities (N = 364) n (%)	Odds Ratio (95% CI)	Univariate p
Female sex	79 (70.5%)	272 (74.7%)	0.81 (0.51–1.31)	0.378
Post-primary education	27 (24.1%)	85 (23.4%)	1.05 (0.63–1.70)	0.869
HIV Virologic Suppression (<200 copies/mL)	107 (95.5%)	348 (95.6%)	0.96 (0.36–3.07)	0.975
History of protease inhibitor exposure	3 (2.7%)	10 (2.7%)	1.01 (0.21–3.43)	0.969
History of abacavir exposure	4 (3.6%)	10 (2.7%)	1.34 (0.35–4.17)	0.652
Obesity	21 (18.8%)	75 (20.6%)	0.89 (0.51–1.51)	0.669
Elevated blood pressure	38 (33.9%)	113 (31.0%)	1.14 (0.72–1.78)	0.566
Hyperglycemia	6 (5.4%)	10 (2.7%)	2.02 (0.66–5.65)	0.180
Self-reported history of hypertension	15 (13.4%)	40 (11.0%)	1.26 (0.65–2.34)	0.486
Self-reported history of diabetes	3 (2.7%)	6 (1.6%)	1.68 (0.33–6.70)	0.484
Self-reported family history of MI or stroke	28 (25.0%)	65 (17.9%)	1.54 (0.92–2.53)	0.095
Current Alcohol Use	52 (46.4%)	182 (50.0%)	0.87 (0.57–1.33)	0.509
Current Tobacco Use	11 (9.8%)	34 (9.3%)	1.07 (0.50–2.13)	0.879
Sedentary lifestyle	36 (32.1%)	118 (32.4%)	0.99 (0.62–1.55)	0.957
Daily fruit and vegetable consumption	9 (8.0%)	50 (13.7%)	0.56 (0.25–1.12)	0.109
	**One or more New ECG Abnormalities (N = 112) mean (sd)**	**No New ECG Abnormalities (N = 364) mean (sd)**	**Univariate p**	
Age, years	45.2 (11.8)	45.8 (10.8)	0.622	
Income, USD^a^	38.82 (47.95)	44.98 (69.75)	0.341	
CD4 (cells/mm^3^)	471.9 (288.1)	492.6 (255.1)	0.497	
Duration of HIV diagnosis, years	5.5 (4.1)	5.7 (4.2)	0.613	
Duration of ART therapy, years	5.1 (3.6)	5.1 (3.7)	0.91	
BMI, kg/m^2^	24.8 (5.1)	25.5 (5.1)	0.183	

^a^Data unavailable for 99 participants

## Discussion

This is one of the first studies to report longitudinal ECG changes among PWH, and to our knowledge, the first such study in SSA. We found that the development of new pathological ECG changes was common over a relatively short 6-month follow-up period, occurring in nearly a quarter of participants. While some of the observed ECG changes may be non-specific, these findings suggest that subclinical CVD is developing in PWH in Tanzania, raising important questions about the need for routine CVD screening in this population.

The most common new ECG abnormalities observed over the follow-up period were T-wave inversions, LVH, ST segment changes, and prolonged QTc interval. T-wave inversions and ST segment changes are non-specific but are often seen in patients with coronary ischemia and heart failure [47–50]. As HIV is a known risk factor for both coronary artery disease and heart failure [4, 6, 7], observing ECG changes suggestive of these pathologies is unsurprising. However, since participants developed these changes over a short period of time, it raises the possibility that subclinical CVD may develop rapidly in this population. While past studies on the side effects of ART have reported longitudinal changes in QTc and PR intervals among PWH in resource-rich settings [26–28], there are few data on other longitudinal ECG changes like new T-wave inversion and ST-segment changes. Thus, additional studies are needed to determine whether PWH in other resource-limited settings experience ECG changes with similar frequency as seen in our study.

LVH was observed in more than 20% of participants at baseline, and an additional 5% of participants developed LVH at six-month follow-up. Several previous studies in Nigeria have shown a higher prevalence of LVH in PWH compared to uninfected controls [31, 33, 34], and an international study on ECG changes in PWH found LVH to be one of the most common ECG abnormalities among its cohort of 7720 PWH [13]. Although LVH has many causes, poorly controlled hypertension is the most common etiology of LVH [51]. Prior research has shown that undiagnosed and uncontrolled hypertension is prevalent among PWH in Tanzania [52], which may explain why LVH was common in our cohort. Our finding suggests that end-organ damage secondary to uncontrolled hypertension may be accumulating rapidly among Tanzanians with HIV. Unfortunately, the management of hypertension and CVD in PWH is currently underdeveloped in sub-Saharan Africa [53, 54]. We recommend that prominent global HIV organizations, such as The Global Fund and The U.S. President’s Emergency Plan for AIDS Relief, consider allocating funds towards strengthening health infrastructure to mitigate the dual burden of HIV and CVD throughout the region. In particular, integrated care for HIV and CVD has shown early promise and may therefore merit investment [55].

Prolonged QTc is associated with fatal ventricular arrhythmias and sudden cardiac death [56]. Although HIV has been associated with prolonged QTc in some settings [30, 32, 57], a prior investigation in Tanzania did not find that prolonged QTc was more common among PWH compared to HIV-uninfected controls [36]. Nonetheless, regular ECG screening in PWH could identify patients with prolonged QTc who are at risk for sudden cardiac death.

This study must be interpreted in light of its limitations. First, we only enrolled individuals actively engaged in HIV care, resulting in a cohort consisting mostly of women with well-controlled HIV; men with uncontrolled HIV were underrepresented in our study. Additionally, excluding participants lost to follow-up may have biased our results in unpredictable ways. This effect is likely minimal, however, since only 4.8% of participants were lost to follow-up. Notably, the ECG findings in this study, such as T wave inversions and resting tachycardia, are non-specific and can be observed in a wide variety of conditions, including non-cardiovascular diseases [58–60]. Finally, this study was conducted over a relatively short 6-month follow up period; future studies are needed to correlate ECG changes with clinically significant CVD outcomes over the longer term. Our exploratory findings require additional investigations with more advanced diagnostic tools, such as echocardiogram and continuous cardiac monitoring.

In conclusion, new non-specific ECG abnormalities such as T-wave inversion, LVH, ST segment changes, and prolonged QTc occur frequently among PWH in Tanzania over a six-month follow-up period. Additional studies are needed to understand the clinical significance of these findings and identify ways to detect subclinical CVD in this high-risk population.

## Supporting information

S1 Table Multivariate associations between participant characteristics and new ECG abnormalities at 6-month follow-up (N = 476). (DOCX)

S1 Data Study data. (XLSX)

S1 File PLOS inclusivity in global research form. (DOCX)

S2 File STROBE statement. (DOCX)
