# Supplementary material for: Longitudinal ECG changes among adults with HIV in Tanzania: A prospective cohort study
Source: PLOS Glob Public Health. 2023 Oct 25;3(10):e0002525. doi: 10.1371/journal.pgph.0002525 (PMC10599566; doi:10.1371/journal.pgph.0002525)
Supplement: S1 Table — (DOCX) [file pgph.0002525.s001.docx]

**S1 Table**: Multivariate Associations between Participant Characteristics and New ECG Abnormalities at 6-Month Follow-Up (N=476)

| **Participant Characteristic** | **One or more New ECG Abnormalities (N=112), n (%)** | **No New ECG Abnormalities (N=364), n (%)** | **Multivariate odds ratio  (95% CI)** | **Multivariate *p*** |
| --- | --- | --- | --- | --- |
| Female sex | 79 (70.5%) | 272 (74.7%) | 0.89 (0.53-1.50) | 0.649 |
| Post-primary education | 27 (24.1%) | 85 (23.4%) | -- | -- |
| HIV Virologic Suppression (<200 copies/mL) | 107 (95.5%) | 348 (95.6%) | -- | -- |
| History of protease inhibitor exposure | 3 (2.7%) | 10 (2.7%) | -- | -- |
| History of abacavir exposure | 4 (3.6%) | 10 (2.7%) | -- | -- |
| Obesity | 21 (18.8%) | 75 (20.6%) | -- | -- |
| Elevated blood pressure | 38 (33.9%) | 113 (31.0%) | -- | -- |
| Hyperglycemia | 6 (5.4%) | 10 (2.7%) | 2.44 (0.71-7.81) | 0.137 |
| Self-reported history of hypertension | 15 (13.4%) | 40 (11.0%) | 1.33 (0.65-2.60) | 0.417 |
| Self-reported history of diabetes | 3 (2.7%) | 6 (1.6%) | 1.13 (0.19-5.51) | 0.881 |
| Self-reported family history of MI or stroke | 28 (25.0%) | 65 (17.9%) | 1.87 (0.60-7.19) | 0.314 |
| Current Alcohol Use | 52 (46.4%) | 182 (50.0%) | -- | -- |
| Current Tobacco Use | 11 (9.8%) | 34 (9.3%) | -- | -- |
| Sedentary lifestyle | 36 (32.1%) | 118 (32.4%) | -- | -- |
| Daily fruit and vegetable consumption | 9 (8.0%) | 50 (13.7%) | 0.51 (0.22-1.05) | 0.085 |
|  |  |  |  |  |
|  | **One or more New ECG Abnormalities (N=112), mean (sd)** | **No New ECG Abnormalities (N=364), mean (sd)** | **Multivariate odds ratio  (95% CI)** | **Multivariate *p*** |
| Age, years | 45.2 (11.8) | 45.8 (10.8) | 0.99 (0.97-1.01) | 0.345 |
| Income, USD^a^ | 38.82 (47.95) | 44.98 (69.75) | -- | -- |
| CD4 (cells/mm^3^) | 471.9 (288.1) | 492.6 (255.1) | 1.00 (1.00-1.00) | 0.439 |
| Duration of HIV diagnosis, years | 5.5 (4.1) | 5.7 (4.2) | -- | -- |
| Duration of ART therapy, years | 5.1 (3.6) | 5.1 (3.7) | -- | -- |
| BMI, kg/m^2^ | 24.8 (5.1) | 25.5 (5.1) | 0.97 (0.93-1.02) | 0.221 |

^a^ Data unavailable for 99 participants
