# Supplementary material for: Longitudinal ECG changes among adults with HIV in Tanzania: A prospective cohort study
Source: PLOS Glob Public Health. 2023 Oct 25;3(10):e0002525. doi: 10.1371/journal.pgph.0002525 (PMC10599566; doi:10.1371/journal.pgph.0002525)
Supplement: S2 File — (DOCX) [file pgph.0002525.s004.docx]

STROBE Statement—checklist of items that should be included in reports of observational studies

|  | Item No. | Recommendation | Page  No. | Relevant text from manuscript |
| --- | --- | --- | --- | --- |
| **Title and abstract** | 1 | (*a*) Indicate the study’s design with a commonly used term in the title or the abstract | 1 | “Prospective Cohort Study” |
|  |  | (*b*) Provide in the abstract an informative and balanced summary of what was done and what was found | 2-3 | Under heading “ABSTRACT” |
| Introduction | | | |  |
| Background/rationale | 2 | Explain the scientific background and rationale for the investigation being reported | 4-5 | “Cardiovascular diseases has become one of the leading causes of…mortality among PWH….no studies have assessed longitudinal ECG changes among PWH in SSA.” |
| Objectives | 3 | State specific objectives, including any prespecified hypotheses | 5 | “we aimed to prospectively describe longitudinal ECG changes over a 6-month period among PWH in Tanzania.” |
| Methods | | | |  |
| Study design | 4 | Present key elements of study design early in the paper | 5-9 | Subsections discuss setting, initial recruitment, and 6-month follow-up, ECG interpretation, statistical analysis, etc. |
| Setting | 5 | Describe the setting, locations, and relevant dates, including periods of recruitment, exposure, follow-up, and data collection | 5-6 | “Moshi, Tanzania”  “Majengo HIV Care and Treatment Clinic”  Enrolled: “September 1, 2020” to “March 1, 2021”  “Six-Month Follow-Up”  Collected survey data, vital signs, health parameters, and 12-lead electrocardiogram. |
| Participants | 6 | (*a*) *Cohort study*—Give the eligibility criteria, and the sources and methods of selection of participants. Describe methods of follow-up  *Case-control study*—Give the eligibility criteria, and the sources and methods of case ascertainment and control selection. Give the rationale for the choice of cases and controls  *Cross-sectional study*—Give the eligibility criteria, and the sources and methods of selection of participants | 5 | “All adults patients (age ≥ 18 years) who presented to MCTC for routine HIV care during the enrolment phase… were eligible for this study. No other exclusion criteria restricted participation.” |
|  |  | (*b*) *Cohort study*—For matched studies, give matching criteria and number of exposed and unexposed  *Case-control study*—For matched studies, give matching criteria and the number of controls per case | N/A | N/A |
| Variables | 7 | Clearly define all outcomes, exposures, predictors, potential confounders, and effect modifiers. Give diagnostic criteria, if applicable | 7-9 | Defined prolonged QTc, left ventricular hypertrophy, bundle branch block, ST elevations, ST depressions, etc. under “ECG Parameter Definitions.” |
| Data sources/ measurement | 8* | For each variable of interest, give sources of data and details of methods of assessment (measurement). Describe comparability of assessment methods if there is more than one group | 8-9 | “Initial and six-month ECGs were interpreted by at least two independent physician adjudicators…” |
| Bias | 9 | Describe any efforts to address potential sources of bias | 8 | “Adjudicators were blinded to all clinical data other than participant age and sex” “In cases of disagreement [on ECG interpretation], a third physician adjudicator served as the tiebreaker.” |
| Study size | 10 | Explain how the study size was arrived at | 9 | “Sample size calculation for this study have been previously reported” [Prattipati et al. cited] |

Continued on next page

| Quantitative variables | 11 | Explain how quantitative variables were handled in the analyses. If applicable, describe which groupings were chosen and why | 9 | “Categorical variables are presented as proportions, and continuous variables as averages with standard deviation.” |
| --- | --- | --- | --- | --- |
| Statistical methods | 12 | (*a*) Describe all statistical methods, including those used to control for confounding | 9 | “Univariate associations between baseline participant characteristics and the presence of any new ECG abnormality at 6-month follow-up were assessed via Pearson’s chi-squared (for categorical variables) and Student’s t-test (for continuous variables).” |
|  |  | (*b*) Describe any methods used to examine subgroups and interactions | 8-9 | “Univariate associations between baseline participant characteristics and the presence of any new ECG abnormality at 6-month follow-up were assessed via Pearson’s chi-squared (for categorical variables) and Student’s t-test (for continuous variables).” |
|  |  | (*c*) Explain how missing data were addressed | 9 | “Participants who received a baseline but not a follow-up ECG were excluded from analysis. Additional missing data were recorded and similarly excluded.” |
|  |  | (*d*) *Cohort study*—If applicable, explain how loss to follow-up was addressed  *Case-control study*—If applicable, explain how matching of cases and controls was addressed  *Cross-sectional study*—If applicable, describe analytical methods taking account of sampling strategy | 9 | “Participants who received a baseline but not a follow-up ECG were excluded from analysis” |
|  |  | (*e*) Describe any sensitivity analyses | N/A | N/A |
| Results | | | | |
| Participants | 13* | (a) Report numbers of individuals at each stage of study—eg numbers potentially eligible, examined for eligibility, confirmed eligible, included in the study, completing follow-up, and analysed | 10 | First paragraph of results and Figure 1 |
|  |  | (b) Give reasons for non-participation at each stage | 10 | “24 participants were lost to follow-up and therefore excluded” |
|  |  | (c) Consider use of a flow diagram | Figure 1 | Figure 1 |
| Descriptive data | 14* | (a) Give characteristics of study participants (eg demographic, clinical, social) and information on exposures and potential confounders | 10-11 | Table 1 |
|  |  | (b) Indicate number of participants with missing data for each variable of interest | 11 | Footnotes under Table 1 |
|  |  | (c) *Cohort study*—Summarise follow-up time (eg, average and total amount) | 11 | 6 month follow-up |
| Outcome data | 15* | *Cohort study*—Report numbers of outcome events or summary measures over time | 12-13 | Tables 2 and 3 |
|  |  | *Case-control study—*Report numbers in each exposure category, or summary measures of exposure |  |  |
|  |  | *Cross-sectional study—*Report numbers of outcome events or summary measures |  |  |
| Main results | 16 | (*a*) Give unadjusted estimates and, if applicable, confounder-adjusted estimates and their precision (eg, 95% confidence interval). Make clear which confounders were adjusted for and why they were included | 13-14 | Table 4 |
|  |  | (*b*) Report category boundaries when continuous variables were categorized | N/A | N/A |
|  |  | (*c*) If relevant, consider translating estimates of relative risk into absolute risk for a meaningful time period | N/A | N/A |

Continued on next page

| Other analyses | 17 | Report other analyses done—eg analyses of subgroups and interactions, and sensitivity analyses | 13-14 | Table 4 |
| --- | --- | --- | --- | --- |
| Discussion | | | | |
| Key results | 18 | Summarise key results with reference to study objectives | 14 | “development of new pathological ECG changes was common over a relatively short 6-month follow-up period…” |
| Limitations | 19 | Discuss limitations of the study, taking into account sources of potential bias or imprecision. Discuss both direction and magnitude of any potential bias | 16 | Second to last paragraph of discussion starting with “This study was be interpreted in light of its limitations.” |
| Interpretation | 20 | Give a cautious overall interpretation of results considering objectives, limitations, multiplicity of analyses, results from similar studies, and other relevant evidence | 14 | “our findings suggest that subclinical CVD is developing in PWH in Tanzania, raising important questions about the need for routine CVD screening in this population.” |
| Generalisability | 21 | Discuss the generalisability (external validity) of the study results | 14-15 | Interpretations are specified to apply to “people with HIV in Tanzania.” |
| Other information | |  | | |
| Funding | 22 | Give the source of funding and the role of the funders for the present study and, if applicable, for the original study on which the present article is based | 1 | “This research was supported by the Duke University Center for AIDS Research (CFAR), an NIH funded program (P30 AI064518), and by Roche Diagnostics. Both grants were awarded to JTH. The funders had no role in study design, data collection and analysis, decision to publish, or preparation of the manuscript.” |

*Give information separately for cases and controls in case-control studies and, if applicable, for exposed and unexposed groups in cohort and cross-sectional studies.

**Note:** An Explanation and Elaboration article discusses each checklist item and gives methodological background and published examples of transparent reporting. The STROBE checklist is best used in conjunction with this article (freely available on the Web sites of PLoS Medicine at http://www.plosmedicine.org/, Annals of Internal Medicine at http://www.annals.org/, and Epidemiology at http://www.epidem.com/). Information on the STROBE Initiative is available at www.strobe-statement.org.
